# Covert Sensing and Communication with Vulnerable Region Control in Near-Field ISAC Systems

**DOI:** 10.3390/s26133976

**Published:** 2026-06-23

**Authors:** Ranhui Xu, Xiaopeng Ji

**Affiliations:** School of Electronic and Information Engineering, Nanjing University of Information Science and Technology, Nanjing 210044, China; 202383270551@nuist.edu.cn

**Keywords:** integrated sensing and communications (ISAC), near-field communication, covert communication, vulnerable region

## Abstract

The deployment of large-scale antenna arrays (ELAAs) in sixth-generation (6G) networks extends wireless communications into the near-field regime, facilitating integrated sensing and communications while introducing security requirements. To ensure secure near-field transmission and sensing accuracy, this paper proposes a framework that jointly minimizes the Cramér–Rao Bound (CRB), guarantees quality-of-service (QoS) for ordinary users, and ensures the covertness of a primary user through an explicit vulnerable-region constraint. The nonconvex problem is addressed through an iterative approach integrating semidefinite relaxation (SDR), alternating optimization (AO), and successive convex approximation (SCA). Numerical results demonstrate sensing performance, QoS satisfaction, and accurate vulnerable-region control.

## 1. Introduction

The demand for massive connectivity and extremely high data rates is driving the move toward sixth-generation (6G) communication systems, where technologies such as extremely large-scale antenna arrays (ELAAs) are gaining increasing attention [1,2]. Large antenna apertures enlarge the near-field regime, under which the planar wave assumption becomes inaccurate and necessitates the adoption of a spherical wave model [3]. This near-field characteristic, in turn, enables precise energy focusing in both distance and angle, a feature highly beneficial for integrated sensing and communication (ISAC) systems [4,5,6]. However, the broadcast nature of wireless channels inherently renders these advanced systems vulnerable to eavesdropping, leading to the rise of covert communication as a vital paradigm beyond traditional physical layer security (PLS) [7]. This paradigm, which is designed to ensure the undetectability of transmissions from a watchful guardian [8,9], is inherently challenging due to fundamental performance limits dictated by the square root law [10]. Therefore, to achieve accurate sensing and high-throughput communication while ensuring the transmission security of near-field ISAC systems, incorporating covert communication into near-field ISAC design is of great significance.

Prior research has laid substantial groundwork in these domains, yet their synergistic integration remains underdeveloped. Although studies on ISAC have focused mainly on balancing performance trade-offs in near-field contexts [4,5,11,12], parallel efforts in covert communication have concentrated on enhancing transmission security through multi-antenna strategies, predominantly in far-field scenarios [13,14,15]. More recent studies have begun to bridge these two research directions. For example, covert communication has been incorporated into ISAC frameworks in far-field environments [16], while the concept of a “vulnerable region” has been introduced to characterize spatial security in near-field systems [17]. In addition, integrated sensing and covert communication architectures have recently been investigated in near-field transmission scenarios, where hybrid beamforming and radar waveform design are jointly optimized to improve covert communication performance while enabling target tracking capabilities [18]. From a broader 6G system perspective, recent work has also discussed ISAC in Frequency Range 3 (FR3) networks under near-field propagation, emphasizing the role of large antenna arrays in joint range and angle estimation, hierarchical beam alignment, and network scale sensing [19]. As ISAC systems become increasingly complex and dynamic, learning-based approaches have attracted growing attention for enabling fast adaptation in time-varying environments and reducing computational complexity [20]. However, these pioneering efforts have primarily focused on single-user or communication-oriented settings, leaving a critical gap in the joint design of resource allocation that can fully exploit near-field propagation characteristics for precise two-dimensional spatial covertness control within a holistic multi-user ISAC framework.

To bridge this critical gap, an optimization scheme for resource allocation in multi-user near-field ISAC systems is developed, aiming to jointly balance sensing precision, service quality for multiple users, and the stringent spatial covertness of a designated primary user (PU). The primary contributions of this work are summarized as follows:

- We propose a new optimization framework for multi-user near-field ISAC to minimize the sensing Cramér–Rao bound (CRB), satisfy quality-of-service (QoS) requirements, and constrain the two-dimensional vulnerable region area to guarantee covert communication for the PU.
- The optimization considered is addressed through an iterative approach based on an analytically tractable model of the warden’s interference power, which combines semidefinite relaxation (SDR), alternating optimization (AO), and successive convex approximation (SCA).
- The simulation results validate the proposed scheme, demonstrating robust sensing, guaranteed QoS, and precise control of vulnerable regions while also revealing the fundamental performance trade-offs.

## 2. System Model

**Figure 1:**
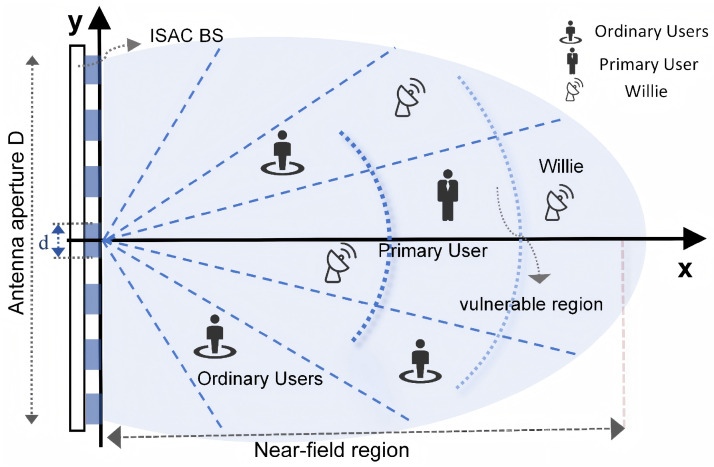
Near-Field ISAC System with Covert Communication.

A near-field ISAC system is investigated in this work, whose architectural configuration is depicted in Figure 1. As illustrated in the figure, a base station (BS) concurrently provides downlink communication to *K* ordinary users (OUs) and one PU while also sensing a single target and ensuring that the PU’s communication remains covert from a warden, Willie. The BS employs a uniform linear array (ULA) consisting of $N=2\tilde{N}+1$ antenna elements spaced by *d*. With the array aperture given by D=(N−1)d, PU and OUs, together with the sensing target, operate within the near-field of BS.

### 2.1. Channel and Signal Model

To accurately describe the near-field propagation geometry, a Cartesian coordinate system is introduced, whose origin is located at the center of the ULA. The position of the *n*-th antenna element is denoted by $s_{n}={\left[(n-\tilde{N})d,\;0\right]}^{T}$, for $n=\{0,1,\ldots ,2\tilde{N}\}$. A user or sensing target is represented by the polar pair (r,θ), from which the distance to the *n*-th antenna element is given by

(1) $$\begin{array}{c} r_{n}\left(r,\theta \right)=\sqrt{r^{2}+{\left(\left(n-\tilde{N}\right)d\right)}^{2}-2r\left(n-\tilde{N}\right)d\cos\theta }, \end{array}$$

Assuming that the loss of the path in free-space is approximately identical for all antenna elements, the complex channel gain is denoted by $\beta =\sqrt{\frac{\lambda_{c}}{4\pi r^{2}}}e^{-j\frac{2\pi }{\lambda_{c}}r}$ [4], where $\lambda_{c}$ is the carrier wavelength. The corresponding phase response across the array is captured by $a\left(r,\theta \right)\in C^{N\times 1}$, whose *n*-th entry is $e^{-j\frac{2\pi }{\lambda_{c}}\left(r_{n}\left(r,\theta \right)-r\right)}$. The near-field communication channel h is determined by the user’s distance *r*, angle θ, and the complex gain β. The resulting channel vector is expressed as

(2) $$\begin{array}{c} h(r,\theta )=\beta a(r,\theta ), \end{array}$$

Let $r_{s},\theta_{s},$ and $\beta_{s}$ denote the distance, angle, and complex channel gain associated with the sensing target, respectively; the near-field sensing response matrix $G\in C^{N\times N}$ is

(3) $$\begin{array}{c} G=\beta_{s}\;a\left(r_{s},\theta_{s}\right)a^{H}\left(r_{s},\theta_{s}\right). \end{array}$$

To jointly serve all users and perform sensing, the BS transmits a superposition of signals. For the PU requiring covert communication, we employ maximum ratio transmission (MRT) beamforming to provide reliable PU communication and enable analytical tractability for covert performance analysis [17]. Let *T* denote the duration of a coherent processing block, $P_{p}$ is the transmit power allocated to the PU, and the downlink transmitted signal $x\left[t\right]\in C^{N\times 1}$ is given by

(4) $$x\left[t\right]=\underset{\mathrm{MRT}\;\mathrm{for}\;\mathrm{PU}}{\underset{︸}{f_{p}c_{p}\left[t\right]}}+\sum_{k=1}^{K}f_{k}c_{k}\left[t\right]+s\left[t\right],$$

where $f_{p}=\sqrt{P_{p}}\;{\bar{a}}_{p}=\frac{\sqrt{P_{p}}}{\sqrt{N}}\frac{\beta_{p}}{|\beta_{p}|}a\left(r_{p},\theta_{p}\right)$ denotes the MRT beamforming vector for the PU, with $\beta_{p}$ being the complex channel gain associated with the PU, $a\left(r_{p},\theta_{p}\right)\in C^{N\times 1}$ being the near-field steering vector toward the PU location $\left(r_{p},\theta_{p}\right)$, and ${\bar{a}}_{p}≜\frac{1}{\sqrt{N}}\frac{\beta_{p}}{|\beta_{p}|}a\left(r_{p},\theta_{p}\right)$ denoting the corresponding normalized MRT direction. The beamformer for OU *k* is denoted by $f_{k}\in C^{N\times 1}$, while s[t] denotes the sensing waveform. Following the near-field ISAC waveform model in [4], the dedicated sensing waveform s[t] is incorporated into the composite transmit signal so that its covariance can be jointly optimized for CRB minimization under the communication QoS constraints. Let the information symbols $c_{p}\left[t\right],c_{k}\left[t\right]$ be uncorrelated with unit power [4]. The covariance matrix associated with x[t], denoted by $R_{x}\in C^{N\times N}$, can be derived as

(5) $$R_{x}=E\left[x\left[t\right]x^{H}\left[t\right]\right]=f_{p}f_{p}^{H}+\sum_{k=1}^{K}f_{k}f_{k}^{H}+R_{s},$$

where $R_{s}\in C^{N\times N}$ denotes the covariance matrix of the sensing waveform. Since the PU beamformer is prescribed by the MRT structure, its covariance component can be written as

(6) $$f_{p}f_{p}^{H}=P_{p}A_{p},$$

where $A_{p}≜{\bar{a}}_{p}{\bar{a}}_{p}^{H}$ is fully determined by the PU location and the corresponding MRT direction. Therefore, the PU-related transmit covariance is parameterized only by the scalar $P_{p}$, rather than being a fully free beamforming covariance matrix. The total transmit power is constrained by $P_{m}$; thus, $\mathrm{tr}\left(R_{x}\right)\leq P_{m}$.

### 2.2. Performance Metrics

#### 2.2.1. Communication QoS

For any generic user u∈{p,1,…,K}, where *p* denotes the PU and 1,…,K denote OUs, the received signal $y_{u}\left[t\right]$ can be defined by

(7) $$y_{u}\left[t\right]=\underset{\mathrm{Desired}\;\mathrm{Signal}}{\underset{︸}{h_{u}^{H}f_{u}c_{u}\left[t\right]}}+\underset{\mathrm{Interference}}{\underset{︸}{h_{u}^{H}\left(\sum_{i\neq u}f_{i}c_{i}\left[t\right]+s\left[t\right]\right)}}+z_{u}\left[t\right],$$

where $h_{u}\in C^{N\times 1}$ is the channel vector to user *u*, $f_{u}$ is its dedicated beamforming vector, $c_{u}\left[t\right]$ is its transmitted symbol, and $z_{u}\left[t\right]∼\mathrm{CN}\left(0,\sigma_{u}^{2}\right)$ is the additive white Gaussian noise (AWGN), while s[t] donates the dedicated sensing signal. The Signal-to-Interference-plus-Noise Ratio (SINR) $\gamma_{u}$ for user *u* is defined as [5]

(8) $$\gamma_{u}=\frac{|h_{u}^{H}f_{u}{|}^{2}}{h_{u}^{H}\left(R_{x}-f_{u}f_{u}^{H}\right)h_{u}+\sigma_{u}^{2}}.$$

From this, the communication rate for user *u* is calculated by $R_{u}=\log_{2}\left(1+\gamma_{u}\right)$.

#### 2.2.2. Sensing Performance

Let $y_{s}\left[t\right]$ denote the sensing waveform collected at the BS, which comprises the signal component reflected by the target and receiver noise $z_{s}\left[t\right]∼\mathrm{CN}\left(0,\sigma_{s}^{2}I_{N}\right)$. The expression for $y_{s}\left[t\right]$ is

(9) $$\begin{array}{c} y_{s}\left[t\right]=Gx\left[t\right]+z_{s}\left[t\right], \end{array}$$

The primary objective is to estimate the target’s distance $r_{s}$ and angle $\theta_{s}$ from the matrix of received echo samples $Y_{s}=\left[y_{s}\left[1\right],\ldots ,y_{s}\left[T\right]\right]$ over the coherent time block. To this end, the sensing performance is characterized by the CRB [11,21], for which the matrix is formulated as

(10) $$\begin{array}{c} \mathrm{CRB}\left(R_{x},G,\sigma_{s}^{2}\right)={\left(J_{11}-J_{12}J_{22}^{-1}J_{12}^{T}\right)}^{-1}, \end{array}$$

Here, $J_{11},J_{12},$ and $J_{22}$ are blocks within the Fisher Information Matrix (FIM) and depend on the transmit covariance matrix $R_{x}$ and the round-trip channel G. The CRB sets the lower bounds on the estimation error variances of the target distance and angle, denoted by $\epsilon_{r_{s}}^{2}$ and $\epsilon_{\theta_{s}}^{2}$, respectively, which are given by

(11) $$\begin{array}{c} \epsilon_{r_{s}}^{2}\geq {\left[\mathrm{CRB}\right]}_{1,1}\;\epsilon_{\theta_{s}}^{2}\geq {\left[\mathrm{CRB}\right]}_{2,2}. \end{array}$$

#### 2.2.3. Vulnerable Region Determination

To explicitly incorporate the PU’s covertness requirement into the spatial design of the considered near-field ISAC system, we characterize spatial covertness through the notion of a vulnerable region, defined as the set of locations at which a potential warden Willie would violate the prescribed covertness requirement if it were located there. Equivalently, it is the spatial region where the received PU signal power at Willie exceeds the admissible covert detection threshold. This subsection aims to derive an explicit boundary and area expression for this region, which will later be used as the covertness metric in Section 3.1. Willie determines whether the PU transmission is present by performing binary hypothesis testing [17]. To this end, we first define the aggregate interference-plus-noise term at Willie as z[t]

(12) $$z\left[t\right]\overset{▵}{=}\sum_{k=1}^{K}h_{w}^{H}f_{k}c_{k}\left[t\right]+h_{w}^{H}s\left[t\right]+n_{w}\left[t\right],$$

where $h_{w}=\beta_{w}a\left(r_{w},\theta_{w}\right)\in C^{N\times 1}$ denotes the near-field channel from the BS to Willie located at $\left(r_{w},\theta_{w}\right)$, with $\beta_{w}$ being the corresponding complex channel gain, and $n_{w}\left[t\right]∼\mathrm{CN}\left(0,\sigma_{w}^{2}\right)$ denotes the AWGN at the single-antenna Willie. The received signal at Willie is then given by

(13) $$y_{w}\left[t\right]=\left\{\begin{array}{cc} z[t], & H_{0},\\ h_{w}^{H}f_{p}c_{p}\left[t\right]+z\left[t\right], & H_{1}, \end{array}\right.$$

Willie performs a binary hypothesis test, where $H_{0}$ corresponds to the case in which only interference and noise are observed, and $H_{1}$ corresponds to the case in which the PU signal is present together with the same interference and noise. Let $D_{1}$ and $D_{0}$ denote the decisions in favor of $H_{1}$ and $H_{0}$, respectively. The false alarm probability and the missed detection probability are defined as $P_{\mathrm{FA}}≜P\left(D_{1}|H_{0}\right)$ and $P_{\mathrm{MD}}≜P\left(D_{0}|H_{1}\right)$, and the corresponding detection error probability is $\xi ≜P_{\mathrm{FA}}+P_{\mathrm{MD}}$. To guarantee covert communication, the detection capability of Willie must be sufficiently limited, which is expressed as $\xi \geq 1-\varepsilon_{c},$ where $\varepsilon_{c}\in \left[0,1\right]$ denotes the prescribed covertness level. According to Pinsker’s inequality [9], the minimum detection error probability of Willie, denoted by $\xi^{*}$, satisfies

(14) $$\xi^{*}\geq 1-\sqrt{\frac{1}{2}D\left(P_{1}\|P_{0}\right)},$$

where $P_{0}$ and $P_{1}$ denote the probability distributions of Willie’s received signal under $H_{0}$ and $H_{1}$, and $D(P_{1}∥P_{0})$ denotes the Kullback–Leibler (KL) divergence between distributions $P_{1}$ and $P_{0}$. Therefore, it is sufficient to ensure that

(15) $$D\left(P_{1}\|P_{0}\right)\leq 2\varepsilon_{c}^{2},$$

The above covertness analysis hinges on the statistical characterization of Willie’s received signal, which depends critically on the aggregate interference term. In the considered multi-user near-field ISAC system, the interference observed by Willie is location-dependent because the OU communication beams and the sensing waveform jointly create a spatially structured radiation pattern. To describe this effect, we define the aggregate covariance matrix of the non-PU transmit components $R_{\mathrm{int}}\in C^{N\times N}$ as

(16) $$R_{\mathrm{int}}≜\sum_{k=1}^{K}f_{k}f_{k}^{H}+R_{s},$$

Then, the warden’s location-dependent interference power can be represented through a normalized spatial focusing coefficient,

(17) $$\eta_{w}\left(r_{w},\theta_{w}\right)≜\frac{a^{H}\left(r_{w},\theta_{w}\right)R_{\mathrm{int}}a\left(r_{w},\theta_{w}\right)}{∥a\left(r_{w},\theta_{w}\right){∥}^{2}\;\mathrm{tr}\left(R_{\mathrm{int}}\right)},$$

which quantifies the effective concentration of the non-PU transmit power at $\left(r_{w},\theta_{w}\right)$ induced by near-field energy focusing. By definition, $\eta_{w}\left(r_{w},\theta_{w}\right)$ is nonnegative and normalized within the interval [0,1] under the adopted normalization. Since $∥a\left(r_{w},\theta_{w}\right){∥}^{2}=N$, the interference plus noise power received at Willie can be expressed as

(18) $$I_{w}\left(r_{w},\theta_{w}\right)={\left|\beta_{w}\right|}^{2}N\mathrm{tr}\left(R_{\mathrm{int}}\right)\eta_{w}\left(r_{w},\theta_{w}\right)+\sigma_{w}^{2}.$$

To preserve analytical tractability, we adopt a region-level scalar approximation for the interference-plus-noise term in Willie’s detection model. Although the noise-only case $I=\sigma_{w}^{2}$ is the most favorable background condition for Willie, the considered multi-user ISAC system necessarily transmits OU communication beams and a dedicated sensing waveform to satisfy the QoS and sensing requirements. Hence, Willie generally observes additional background energy generated by the non-PU components. Since the exact interference plus noise power $I_{w}\left(r_{w},\theta_{w}\right)$ varies with Willie’s location due to the spatially varying near-field radiation pattern, directly retaining it would couple the covertness boundary with the non-PU transmit covariance and prevent a closed-form expression of the vulnerable-region area $S_{v}$. Therefore, following the path-loss approximation adopted in [17], the large-scale channel gain $|\beta_{w}{|}^{2}$ is evaluated according to the PU communication range and treated as a fixed reference value in the boundary derivation. Under the transmit power constraint, the non-PU transmit power satisfies $\mathrm{tr}\left(R_{\mathrm{int}}\right)\leq P_{m}-P_{p}$. Together with $\eta_{w}\left(r_{w},\theta_{w}\right)\leq 1$, the adopted scalar approximation is given by $I≜|\beta_{w}{|}^{2}N\left(P_{m}-P_{p}\right)+\sigma_{w}^{2}$. This scalar approximation retains the masking effect of the unavoidable OU and sensing transmissions while replacing the location-dependent $I_{w}\left(r_{w},\theta_{w}\right)$ with a tractable analytical quantity rather than a point-wise exact interference guarantee. It converts the covertness condition into a beampattern threshold [16], thereby enabling the closed-form derivation of $S_{v}$. Under the above model, Willie’s received signal under $H_{0}$ and $H_{1}$ follows zero-mean complex Gaussian distributions with powers $\lambda_{0}=I$ and $\lambda_{1}={\left|h_{w}^{H}f_{p}\right|}^{2}+I$, respectively. Therefore, over the coherent time block of length *T*, the KL divergence in (14) is given by

(19) $$D\left(P_{1}∥P_{0}\right)=T\left(\frac{\lambda_{1}}{\lambda_{0}}-\ln\frac{\lambda_{1}}{\lambda_{0}}-1\right).$$

Defining $\nu ≜\frac{|h_{w}^{H}f_{p}{|}^{2}}{I}$, the above expression can be rewritten as

(20) $$D\left(P_{1}∥P_{0}\right)=T\left[\nu -\ln\left(1+\nu \right)\right].$$

In the covert regime, ν is extremely small, and thus the approximation $\nu -\ln\left(1+\nu \right)\approx \nu^{2}/2$ can be applied. Substituting this approximation into (15), the covertness requirement [17] can be expressed as

(21) $$|h_{w}^{H}f_{p}{|}^{2}\leq \frac{2\varepsilon_{c}}{\sqrt{T}}\left[|\beta_{w}{|}^{2}N\left(P_{m}-P_{p}\right)+\sigma_{w}^{2}\right]≜Q.$$

where *Q* denotes the covert power threshold. To translate this power threshold into a spatial geometric boundary, we first introduce the normalized threshold $Q_{n}$:

(22) $$Q_{n}\overset{▵}{=}\frac{Q}{P_{p}{\left|\beta_{w}\right|}^{2}N},$$

The near-field beampattern power B(r,θ) of the PU array, characterizing the spatial power distribution [22], is given by

(23) $$B\left(r,\theta \right)=\frac{|a^{H}\left(r,\theta \right)\;a\left(r_{p},\theta_{p}\right){|}^{2}}{N^{2}}.$$

To further obtain the radial boundary in closed form, we focus on the mainlobe direction by setting $\theta =\theta_{p}$. By applying a second-order distance expansion and a Fresnel approximation, the PU beampattern along $\theta_{p}$ can be approximated as

(24) $$\sqrt{B(r,\theta_{p})}\approx \left|\frac{C(u)+jS(u)}{u}\right|,$$

where C(u) and S(u) are the Fresnel integrals defined by $C\left(u\right)=\int_{0}^{u}\cos\left(\frac{\pi t^{2}}{2}\right)dt,S\left(u\right)=\int_{0}^{u}\sin\left(\frac{\pi t^{2}}{2}\right)dt,$ and $u=\sqrt{\frac{N^{2}d^{2}\cos^{2}\theta_{p}}{2\lambda_{c}}\left|\frac{1}{r}-\frac{1}{r_{p}}\right|}$ [17]. Since the covertness constraint requires that the PU signal power observed at any potential warden location should not exceed the normalized threshold $Q_{n}$, and this received power is characterized by the beampattern $B(r,\theta_{p})$ along the mainlobe direction, the covertness requirement can be written as

(25) $$B\left(r,\theta_{p}\right)\leq Q_{n}.$$

Substituting the above equation into (24) gives $\left|\frac{C(u)+jS(u)}{u}\right|\leq \sqrt{Q_{n}}.$ At the boundary of the vulnerable region, the above inequality holds with equality, namely $\left|\frac{C\left(u_{0}\right)+jS\left(u_{0}\right)}{u_{0}}\right|=\sqrt{Q_{n}}$, where $u_{0}$ denotes the positive root associated with the covertness threshold. This boundary condition is equivalently written as $\left|\frac{1}{r}-\frac{1}{r_{p}}\right|=\frac{2\lambda_{c}u_{0}^{2}}{N^{2}d^{2}\cos^{2}\theta_{p}}≜Z_{\Delta }$. Hence, the two solutions of the above equation determine the lower and upper radial boundaries of the vulnerable region, namely $r_{\mathrm{Low}}={\left(1/r_{p}+Z_{\Delta }\right)}^{-1}$ and $r_{\mathrm{Up}}={\left(1/r_{p}-Z_{\Delta }\right)}^{-1}$. Finally, the vulnerable-region area $S_{v}$ is defined as

(26) $$S_{v}=\frac{\Delta \theta }{2}\left[{\left(\frac{1}{\frac{1}{r_{p}}-Z_{\Delta }}\right)}^{2}-{\left(\frac{1}{\frac{1}{r_{p}}+Z_{\Delta }}\right)}^{2}\right].$$

Here, $\Delta \theta =\arcsin\left(\frac{2}{N}\right)$ is the angular extent of the vulnerable region, determined by the array’s minimum angular resolution.

## 3. Problem Formulation and Solutions

### 3.1. Problem Formulation

In this section, we formulate an optimization problem that minimizes the CRB associated with estimating the target range $r_{s}$ and direction $\theta_{s}$. The design enforces per-user QoS requirements while limiting the area of the vulnerable region. For this optimization, the target’s position ($r_{s},\theta_{s}$) is assumed to be fixed [11]. The optimization problem is stated as follows:

(27a) $$\begin{array}{cc} \min_{f_{p},{\left\{f_{k}\right\}}_{k=1}^{K},R_{s}}\; & \mathrm{tr}(\mathrm{CRB}\left(R_{x},G,\sigma_{s}^{2}\right)) \end{array}$$

(27b) $$\begin{array}{cc} s.t.\; & \mathrm{tr}\left(R_{x}\right)\leq P_{m} \end{array}$$

(27c) $$\begin{array}{cc} \; & \gamma_{k}\left(R_{x},f_{k}\right)\geq \gamma_{\min,k},\;\forall k \end{array}$$

(27d) $$\begin{array}{cc} \; & \gamma_{p}\left(R_{x},f_{p}\right)\geq \gamma_{\min,p} \end{array}$$

(27e) $$\begin{array}{cc} \; & S_{v}\left(f_{p},{\left\{f_{k}\right\}}_{k=1}^{K},R_{x}\right)\leq \epsilon \end{array}$$

(27f) $$\begin{array}{cc} \; & R_{s}⪰0 \end{array}$$

where constraint (27b) enforces the total transmit power budget $P_{m}$, constraints (27c) and (27d) ensure the minimum QoS requirements for the OUs and the PU, and constraint (27e) restricts the vulnerable-region area $S_{v}$ to be below a predefined threshold ϵ. Since the PU beamformer is prescribed by MRT, the proposed formulation optimizes the PU transmission under an MRT-constrained architecture rather than a fully flexible beamforming design. Although MRT may be suboptimal compared with a fully optimized PU beamformer, it is adopted due to its closed-form structure and the resulting tractable CRB-oriented design. Moreover, MRT maximizes the received signal power along the PU channel direction, offering a low-complexity and effective transmission strategy. Therefore, it provides a reasonable tradeoff between beamforming optimality and analytical tractability. A brief comparison with a fully optimized PU beamformer to illustrate the performance gap will be provided in Section 4.2.

### 3.2. Problem Solution

To address the non-convexity of problem (Equation (27a–f)) due to quadratic forms of beamforming vectors, we introduce matrix variables for notational convenience. In particular, for the PU, its covariance matrix is retained in the structured form induced by the prescribed MRT beamformer, whereas for the OUs, SDR is applied to relax the beamforming outer products [4]. Specifically, we define

(28) $$\begin{array}{c} F_{p}≜f_{p}f_{p}^{H}=P_{p}A_{p},\;F_{k}≜f_{k}f_{k}^{H},\;\forall k. \end{array}$$

where $A_{p}$ is the normalized beamforming matrix defined in (6). Here, $F_{p}\in C^{N\times N}$ inherently remains rank-one due to the PU’s MRT-determined $f_{p}$, while the non-convex rank-one constraint on $F_{k}\in C^{N\times N}$ for OUs is relaxed via SDR. After this relaxation, the objective function and constraints (27c), (27d), and (27e) from problem (Equation (27a–f)) remain non-convex, with rank-one recovery for OUs performed using standard techniques [12].

To further handle the remaining non-convexity after SDR, we develop a hybrid AO-SCA algorithm in Section 3.2.1 that alternates variable updates and convexifies the non-convex terms via first-order Taylor approximations. The efficiency of this approach relies on accurate linearizations, which are provided in Section 3.2.2 through the analytically derived gradients of the SINR and the numerically evaluated first-order approximations for the CRB objective and the vulnerable-region area.

#### 3.2.1. A Hybrid AO-SCA Approach

An AO framework integrated with SCA is employed for the SDR-relaxed problem, which decomposes the optimization into three sequentially solved subproblems. In iteration *j*, given the solution from iteration j−1, $F_{p}^{\left(j-1\right)},{\left\{F_{k}^{\left(j-1\right)}\right\}}_{k=1}^{K},R_{s}^{\left(j-1\right)}$, which serves as a linearization point, the following subproblems are solved sequentially. Applying first-order SCA, the non-convex terms are linearized around the solution from iteration j−1, and the resulting approximated objective functions and constraints are denoted with a tilde (∼), where $\nabla_{R_{x}}\mathrm{CRB}\left(R_{x}^{\left(j-1\right)}\right)$ represents the gradient of the CRB with respect to $R_{x}$ evaluated at the point $R_{x}^{\left(j-1\right)}$.

**Subproblem 1: Optimization of** $F_{p}$

To simplify the notation, we first define the constant term $\Theta_{1}^{\left(j-1\right)}=\sum_{k=1}^{K}F_{k}^{\left(j-1\right)}+R_{s}^{\left(j-1\right)}$. Recalling that the PU covariance matrix is restricted to the MRT-induced structured form $F_{p}=P_{p}A_{p}$, the corresponding subproblem is formulated as

(29a) $$\begin{array}{cc} \min_{F_{p}⪰0}\;\; & \mathrm{tr}\;\left(\nabla_{R_{x}}\mathrm{CRB}\left(R_{x}^{\left(j-1\right)}\right)\cdot \left(F_{p}+\Theta_{1}^{\left(j-1\right)}\right)\right) \end{array}$$

(29b) $$\begin{array}{cc} s.t.\;\; & F_{p}=P_{p}A_{p},\;\;P_{p}\geq 0 \end{array}$$

(29c) $$\begin{array}{cc} \; & \mathrm{tr}\left(F_{p}+\Theta_{1}^{\left(j-1\right)}\right)\leq P_{m} \end{array}$$

(29d) $$\begin{array}{cc} \; & {\tilde{\gamma }}_{p}\left(F_{p};F_{p}^{\left(j-1\right)}\right)\geq \gamma_{\min,p} \end{array}$$

(29e) $$\begin{array}{cc} \; & {\tilde{S}}_{v}\left(F_{p};F_{p}^{\left(j-1\right)},\Theta_{1}^{\left(j-1\right)}\right)\leq \epsilon \end{array}$$

**Subproblem 2: Sequential optimization of** $F_{k}$

With the structured PU covariance matrix $F_{p}$ and the sensing covariance matrix $R_{s}$ fixed, the OU beamforming covariance matrices are updated sequentially. Specifically, when updating $F_{k}$, the covariance matrices of all other OUs are fixed at their most recently available values. For notational convenience, we define $\Theta_{2}^{\left(j\right)}=F_{p}^{\left(j\right)}+R_{s}^{\left(j-1\right)}+\sum_{i\neq k}F_{i}$. Then, $F_{k}$ is updated by solving

(30a) $$\begin{array}{cc} \min_{F_{k}⪰0}\;\; & \mathrm{tr}\left(\nabla_{R_{x}}\mathrm{CRB}\left(R_{x}^{\left(j-1\right)}\right)\cdot \left(\Theta_{2}^{\left(j\right)}+F_{k}\right)\right) \end{array}$$

(30b) $$\begin{array}{cc} s.t.\;\; & \mathrm{tr}\left(\Theta_{2}^{\left(j\right)}+F_{k}\right)\leq P_{m} \end{array}$$

(30c) $$\begin{array}{cc} \; & {\tilde{\gamma }}_{k}\left(F_{k};F_{k}^{\left(j-1\right)}\right)\geq \gamma_{\min,k} \end{array}$$

(30d) $$\begin{array}{cc} \; & {\tilde{S}}_{v}\left(F_{k};F_{p}^{\left(j\right)},\Theta_{2}^{\left(j\right)}\right)\leq \epsilon \end{array}$$

**Subproblem 3: Optimization of** $R_{s}$

With the structured PU covariance matrix $F_{p}$ and ${\left\{F_{k}\right\}}_{k=1}^{K}$ fixed at their latest values, the sensing signal covariance matrix $R_{s}$ is updated by

(31a) $$\begin{array}{cc} \min_{R_{s}⪰0}\; & \mathrm{tr}(\nabla_{R_{x}}\mathrm{CRB}\left(R_{x}^{\left(j-1\right)}\right)\cdot \left(F_{p}^{\left(j\right)}+F_{k}^{\left(j\right)}+R_{s}\right)) \end{array}$$

(31b) $$\begin{array}{cc} s.t.\; & \mathrm{tr}\left(F_{p}^{\left(j\right)}+F_{k}^{\left(j\right)}+R_{s}\right)\leq P_{m} \end{array}$$

(31c) $$\begin{array}{cc} \; & {\tilde{S}}_{v}\left(R_{s};F_{p}^{\left(j\right)},F_{k}^{\left(j\right)}\right)\leq \epsilon \end{array}$$

During iteration *j*, each optimization variable is refined via successive convex approximation, where the original non-convex expressions are replaced by their linear surrogates constructed at the prior iterate.

Specifically, the CRB objective function is approximated by its first-order Taylor expansion with respect to the transmit covariance matrix $R_{x}$ evaluated at $R_{x}^{\left(j-1\right)}$. This approximation results in a linear objective function in the current optimization variables. For the communication quality of service constraints, the original SINR expressions are replaced by their first-order affine approximations, denoted by ${\tilde{\gamma }}_{p}\left(\cdot \right)$ and ${\tilde{\gamma }}_{k}\left(\cdot \right)$, which are locally tight at the corresponding linearization points. Similarly, the vulnerable-region constraint is approximated by the surrogate function ${\tilde{S}}_{v}\left(\cdot \right)$, which represents the first-order linearization of the vulnerable-region area $S_{v}$ around the current linearization point.

With these surrogate functions, all nonlinear components are transformed into affine expressions. Together with the trace constraints, the semidefinite constraints on $F_{k}$ and $R_{s}$, and the structured PU covariance constraint $F_{p}=P_{p}A_{p}$, each subproblem becomes a convex semidefinite program (SDP) that can be efficiently solved by CVX. The corresponding gradients used to construct these first-order surrogates are detailed next.

#### 3.2.2. Gradient Derivations for SCA

To construct the first-order surrogate functions introduced in Section 3.2.1, this subsection derives the gradients required for the CRB objective, the SINR constraints, and the vulnerable-region constraint. Specifically, at the (j−1)-th iteration, a differentiable scalar function f(X) can be approximated around $X^{\left(j-1\right)}$ as

(32) $$\begin{array}{c} \tilde{f}\left(X;X^{\left(j-1\right)}\right)=f\left(X^{\left(j-1\right)}\right)+\mathrm{tr}\left(\nabla_{X}f{\left(X^{\left(j-1\right)}\right)}^{H}\left(X-X^{\left(j-1\right)}\right)\right). \end{array}$$

which is used throughout the AO-SCA procedure. In particular, the CRB term is linearized with respect to the overall transmit covariance matrix $R_{x}$, whereas the SINR and $S_{v}$ terms are linearized with respect to the optimization block currently being updated. To this end, the corresponding gradients are derived in the following.

**Gradient of SINR:** The SINR functions $\gamma_{p}\left(F_{p}\right)$ and $\gamma_{k}\left(F_{k}\right)$ admit closed-form expressions, and their first-order affine approximations are constructed from exact analytical gradients obtained by matrix calculus. For PU, the SINR can be written as $\gamma_{p}\left(F_{p}\right)=\frac{h_{p}^{H}F_{p}h_{p}}{{\mathrm{Denom}}_{p}}$, where ${\mathrm{Denom}}_{p}=h_{p}^{H}\left(\sum_{i\neq p}F_{i}+R_{s}\right)h_{p}+\sigma_{p}^{2}$. Similarly, for OU *k*, when $F_{k}$ is updated sequentially, the SINR can be written as $\gamma_{k}\left(F_{k}\right)=\frac{h_{k}^{H}F_{k}h_{k}}{{\mathrm{Denom}}_{k}}$, where ${\mathrm{Denom}}_{k}=h_{k}^{H}\left(F_{p}+\sum_{i\neq k}F_{i}+R_{s}\right)h_{k}+\sigma_{k}^{2}$. When $F_{p}$ or $F_{k}$ is updated, the covariance matrices appearing in the corresponding denominator are fixed at their most recently available values. Therefore, ${\mathrm{Denom}}_{p}$ and ${\mathrm{Denom}}_{k}$ are independent of the current optimization variables $F_{p}$ and $F_{k}$, respectively, and the first-order approximation uses the gradients with respect to the corresponding numerator variables:

(33) $$\begin{array}{c} \nabla_{F_{p}}\gamma_{p}=\frac{h_{p}h_{p}^{H}}{{\mathrm{Denom}}_{p}},\;\nabla_{F_{k}}\gamma_{k}=\frac{h_{k}h_{k}^{H}}{{\mathrm{Denom}}_{k}}. \end{array}$$

Substituting these gradients into (32) gives the affine lower bounds ${\tilde{\gamma }}_{p}\left(\cdot \right)$ and ${\tilde{\gamma }}_{k}\left(\cdot \right)$ used in (29d) and (30c), respectively.

**Gradient of the CRB and Vulnerable-Region Area:** Accurate analytical gradient derivation for the objective function of the CRB and the area of the vulnerable region $S_{v}$ presents significant challenges due to complex matrix inversions in (10) and transcendental Fresnel integrals and implicit boundaries defining $S_{v}$ in (23). Therefore, the gradients for both are numerically approximated at each iteration using the finite central-difference method [23].

For the CRB objective, let F(X)=tr(CRB(X)). Its (m,n)-th gradient element is approximated by

(34) $$\nabla_{R_{x}}F\approx \frac{F\;\left(R_{x}^{\left(j-1\right)}+\delta E_{m,n}\right)-F\;\left(R_{x}^{\left(j-1\right)}-\delta E_{m,n}\right)}{2\delta }.$$

Here, $E_{m,n}$ denotes an N×N perturbation matrix whose (m,n)-th entry equals one and all other entries are zero, and δ is a small perturbation step used for numerical gradient estimation. According to the first-order approximation in (32), the CRB objective is linearized with respect to $R_{x}$. In the subproblem formulations (29a), (30a) and (31a), the constant terms in the Taylor expansion are omitted, since they are independent of the current optimization variable and thus do not affect the optimal solution.

For $S_{v}$, the scalar partial derivatives with respect to $P_{p}$ and $P_{\mathrm{int}}$ are approximated by

(35) $$\begin{array}{cc} \frac{\partial S_{v}}{\partial P_{p}} & \approx \frac{S_{v}\left(P_{p}+\Delta P_{p},P_{\mathrm{int}}\right)-S_{v}\left(P_{p}-\Delta P_{p},P_{\mathrm{int}}\right)}{2\Delta P_{p}} \end{array}$$

(36) $$\begin{array}{cc} \frac{\partial S_{v}}{\partial P_{\mathrm{int}}} & \approx \frac{S_{v}\left(P_{p},P_{\mathrm{int}}+\Delta P_{\mathrm{int}}\right)-S_{v}\left(P_{p},P_{\mathrm{int}}-\Delta P_{\mathrm{int}}\right)}{2\Delta P_{\mathrm{int}}} \end{array}$$

where $P_{\mathrm{int}}=\sum_{k=1}^{K}\mathrm{tr}\left(F_{k}\right)+\mathrm{tr}\left(R_{s}\right)$ represents the aggregate interference power generated by the OU communication beams and the sensing waveform. Then, by applying the chain rule and recalling the MRT-induced structured form $F_{p}=P_{p}A_{p}$, the gradients of $S_{v}$ with respect to $F_{p},F_{k},$ and $R_{s}$ are given by

(37) $$\begin{array}{c} \nabla_{F_{p}}S_{v}=\frac{\partial S_{v}}{\partial P_{p}}A_{p},\;\nabla_{F_{k}}S_{v}=\nabla_{R_{s}}S_{v}=\frac{\partial S_{v}}{\partial P_{\mathrm{int}}}I_{N}. \end{array}$$

where $I_{N}$ is the N×N identity matrix. By substituting these gradients into the first-order approximation in (32), the affine surrogate functions for the vulnerable-region constraint are constructed and used in (29e), (30d), and (31c).

Since the gradients in (34), (35), and (36) are obtained via finite-difference approximations, their evaluation introduces a tradeoff between approximation accuracy and computational complexity. Accordingly, the perturbation parameters δ, $\Delta P_{p}$, and $\Delta P_{\mathrm{int}}$ affect both the convergence behavior and the final performance, and their impact is examined in Section 4.1.

Based on the above AO-SCA framework, the proposed algorithm solves a sequence of SDR-relaxed convex subproblems and updates the transmit covariance variables iteratively. Since each subproblem is solved with locally tight first-order approximations around the current iterate, the obtained solution approaches a stationary point of the SDR-relaxed problem. Since the SDR-relaxed problem remains non-convex after decomposition, the proposed AO-SCA algorithm may still depend on the feasible initialization and the block update trajectory. Therefore, the obtained solution is interpreted as a stationary solution under the prescribed fixed update order, rather than as a globally optimal solution. In the simulations, the same deterministic update protocol is used for all compared settings, and the observed convergence behavior verifies the stability of the proposed procedure under the considered system parameters. Following the standard interior point complexity estimate for SDP solvers [24,25], the computational complexity of the proposed hybrid AO-SCA algorithm is mainly determined by the convex SDP subproblems in (Equations (29a–e)–(31a–c)) and the finite central difference calculations in (34)–(36). The dominant numbers of complex multiplications and additions in one AO iteration are summarized in Table 1.

In Table 1, the term $O(N^{3.5})$ follows from the standard interior point complexity estimate for solving an SDP problem with one N×N semidefinite variable and a fixed number of affine constraints [25]. Since the PU covariance matrix is restricted to the MRT-induced form $F_{p}=P_{p}A_{p}$, (Equation (29a–e)) only optimizes the scalar variable $P_{p}$ and does not contain an N×N SDP solver term. The OU update entry in Table 1 refers to the update of a single OU covariance matrix $F_{k}$. Since the row of each OU update in Table 1 is repeated *K* times in each AO iteration, the covariance summations and problem assembly operations in the sequential OU updates contribute an additional cost of $O(K^{2}N^{2})$. Therefore, if $I_{\mathrm{AO}}$ denotes the number of AO iterations required for convergence, the overall computational complexity is given by $O\left(I_{\mathrm{AO}}\left(N^{4}+KN^{3.5}+N^{3.5}+K^{2}N^{2}\right)\right)$, where the terms $N^{4}$, $KN^{3.5}$, $N^{3.5}$, and $K^{2}N^{2}$ correspond to the finite central difference CRB gradient calculation, the *K* sequential OU SDP updates, the sensing covariance SDP update, and the covariance summations and problem assembly operations, respectively.

## 4. Numerical Results

**Figure 2:**
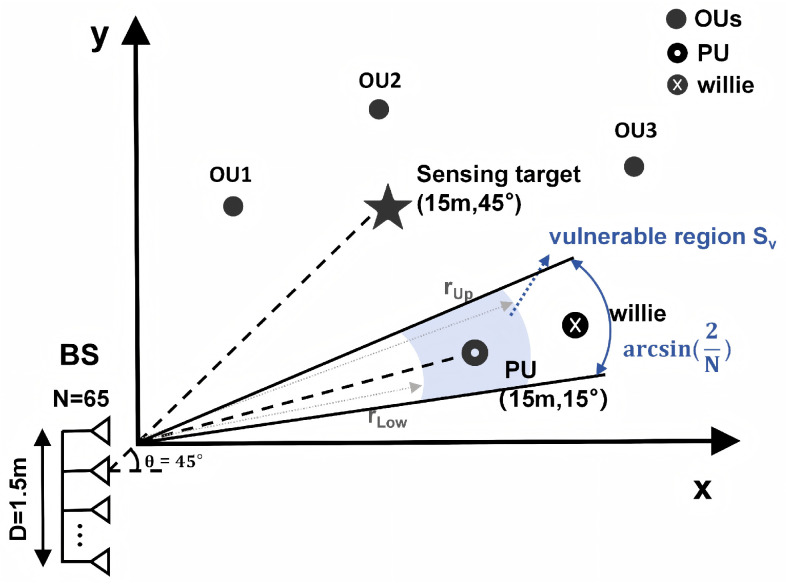
Simulation layout and geometric illustration of the vulnerable region.

In this section, numerical results are presented to assess the performance of the proposed framework. Our simulation model considers a ULA of N=65 elements deployed in the BS, spanning an aperture of D=1.5m and operating at $f_{c}=56\;\mathrm{GHz}$ ($\lambda_{c}\approx 0.536\;\mathrm{cm}$). The total transmit power of the BS is set to $P_{m}=20\;\mathrm{dBm}$, and AWGN noise power at all communication receivers and Willie is $\sigma^{2}=-60\;\mathrm{dBm}$. Signal transmission spans a coherent time block of T=100 snapshots, and the prescribed covertness level is set to $\varepsilon_{c}=0.01$. The system includes K=3 OUs, one PU, and a single sensing target, all operating in the BS near-field region. The PU position is assumed to be fixed at $\left(r_{p},\theta_{p}\right)=\left(15\;m,15{}^{\circ}\right)$, while the OUs are randomly distributed in the coverage area. The sensing target is located at $\left(r_{s},\theta_{s}\right)=\left(15\;m,45{}^{\circ}\right)$. Figure 2 shows the simulation layout and the related geometric characteristics considered in the numerical results. The perturbation parameters used for numerical gradient estimation are set to δ=0.01 and $\Delta P_{p}=\Delta P_{\mathrm{int}}=1\times 10^{-3}$ W.

### 4.1. Convergence Performance of the Proposed AO-SCA Algorithm

**Figure 3:**
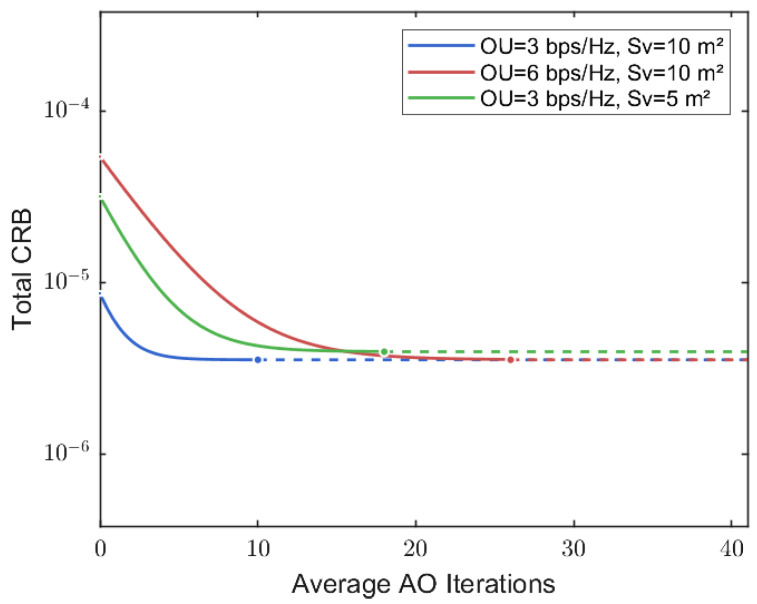
Convergence of the Total CRB (trace of the CRB matrix) versus average AO iterations under varying QoS and covertness constraints.

Figure 3 illustrates the convergence behavior of the Total CRB achieved by the proposed AO-SCA algorithm, averaged over multiple Monte Carlo realizations with a fixed minimum PU communication rate of 5 bps/Hz. Here, the Total CRB refers to the trace of the CRB matrix [26], obtained by summing the diagonal CRB entries associated with the target distance and angle parameters. It is used as a compact aggregate metric to reflect the overall sensing accuracy, where a smaller value indicates a lower joint estimation error bound. The simulation results indicate that lower OU communication rates or less stringent vulnerable region area requirements lead to a lower initial Total CRB and faster convergence. Conversely, higher OU communication rates or more stringent $S_{v}$ requirements result in higher initial Total CRB values and slower convergence. Notably, within a practical range of operational parameters, the converged Total CRB values across different constraint combinations exhibit minimal variation.

Since the proposed AO-SCA algorithm relies on numerically evaluated gradients for the CRB and vulnerable-region terms, we further examine the sensitivity of convergence and final performance to the perturbation parameters used in the finite-difference approximation.

To further evaluate the perturbation parameters used in the numerical gradient approximation, Table 2 reports the average AO iterations and the final Total CRB for the same representative setting shown by the red curve in Figure 3. The results show that the final Total CRB changes only slightly within the tested range, with a maximum relative change of 2.26%, whereas the convergence speed is more sensitive to the parameter choice. Smaller perturbation values lead to slower convergence, while larger perturbation values reduce the iteration number at the cost of a slightly higher final Total CRB. Hence, the choice δ=0.01 and $\Delta P_{p}=\Delta P_{\mathrm{int}}=1\times 10^{-3}$ W achieves a good balance between convergence efficiency and approximation accuracy, indicating reasonable robustness of the adopted numerical gradient implementation.

### 4.2. RCRB Versus Minimum OUs Communication Rate

**Figure 4:**
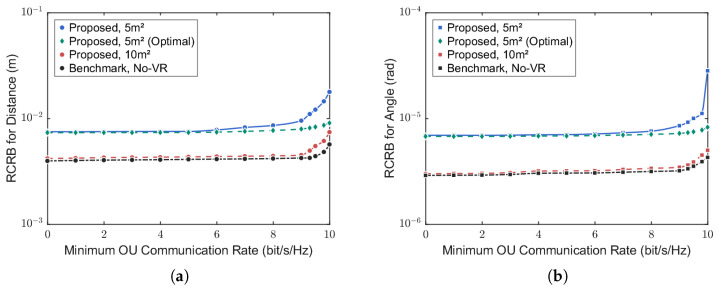
RCRB versus minimum OU communication rate under different vulnerable region constraints, with the PU rate fixed at 5 bps/Hz: (**a**) distance estimation and (**b**) angle estimation.

To evaluate the impact of the vulnerable region area constraint and the PU beamforming structure on sensing performance, we compare the RCRB for distance and angle estimation under different OU communication rate requirements. Here, the RCRB refers to the square root of the corresponding diagonal CRB entry in (11), and therefore characterizes the lower bound on the root mean square estimation error of each parameter. Specifically, we consider two vulnerable region area constraints, i.e., $S_{v}=5\;m^{2}$ and $S_{v}=10\;m^{2}$, and include a benchmark without the vulnerable region constraint to show the limited performance loss caused by introducing the proposed vulnerable region framework. In addition, for $S_{v}=5\;m^{2}$, we compare the MRT-based PU beamformer with a fully optimized PU beamformer. The PU rate is fixed at 5 bps/Hz. The results are shown in Figure 4, where Figure 4a,b correspond to distance and angle estimation, respectively. It can be observed that the RCRB for both distance and angle estimation increase as the minimum OU communication rate becomes larger, especially in the high-rate region. Since a smaller RCRB corresponds to a higher estimation accuracy, this indicates that a more stringent OU communication requirement degrades both distance and angle sensing accuracy in the considered setup. Moreover, the curves corresponding to $S_{v}=5\;m^{2}$ are generally higher than those for $S_{v}=10\;m^{2}$, indicating that a smaller vulnerable region area constraint results in larger RCRB and thus lower distance and angle estimation accuracy. The benchmark without the vulnerable region constraint achieves the lowest RCRBs among the compared schemes, while the proposed design with the vulnerable region area constraint exhibits only a small performance loss in terms of distance and angle estimation accuracy. For the two curves with $S_{v}=5\;m^{2}$, the fully optimized PU beamformer achieves a slightly lower RCRB than the MRT-based design. When the OU communication rate requirement is relatively loose, the two curves are close to each other, indicating that the MRT-based PU beamformer can achieve comparable estimation accuracy to the fully optimized design in this region. Since the MRT structure fixes the PU beamforming direction, it can reduce the computational complexity of solving the resulting optimization problem while maintaining comparable sensing performance.

### 4.3. Sensing Performance and Covertness Versus PU Angle

**Figure 5:**
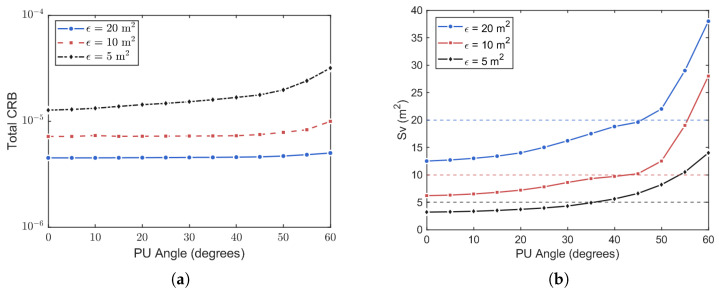
Sensing performance and covertness versus PU angle under different covertness constraints ϵ: (**a**) sensing performance (Total CRB) versus PU angle; (**b**) covertness performance ($S_{v}$) versus PU angle.

To evaluate the impact of the PU angle on sensing performance and the vulnerable region area, we vary the PU azimuth angle $\theta_{p}$ while fixing the PU distance as 15 m. The minimum rate requirements of both the PU and OUs are set as 5 bps/Hz. We consider three vulnerable region area constraints, i.e., $\epsilon =20\;m^{2}$, $\epsilon =10\;m^{2}$, and $\epsilon =5\;m^{2}$. The results are shown in Figure 5, where Figure 5a shows the Total CRB and Figure 5b shows the corresponding vulnerable region area $S_{v}$. In Figure 5a, it can be observed that the Total CRB remains relatively stable when $\theta_{p}$ is small or moderate, and increases when $\theta_{p}$ becomes large, indicating degraded sensing accuracy at large PU angles. In addition, for the same PU angle, a smaller ϵ leads to a larger Total CRB, indicating lower sensing accuracy under stricter vulnerable region area constraints. Figure 5b shows that the vulnerable region area $S_{v}$ increases with $\theta_{p}$, and the increase becomes more obvious at large PU angles. The intersection between each $S_{v}$ curve and the corresponding constraint level indicates the largest PU angle that can be supported. It can be observed that the allowable PU angle range under $\epsilon =5\;m^{2}$ is smaller than that under $\epsilon =10\;m^{2}$, while $\epsilon =20\;m^{2}$ allows the largest range, indicating that a stricter covertness requirement limits the feasible PU angle range in this simulation setting.

### 4.4. Sensing Performance Versus Willie Distance

**Figure 6:**
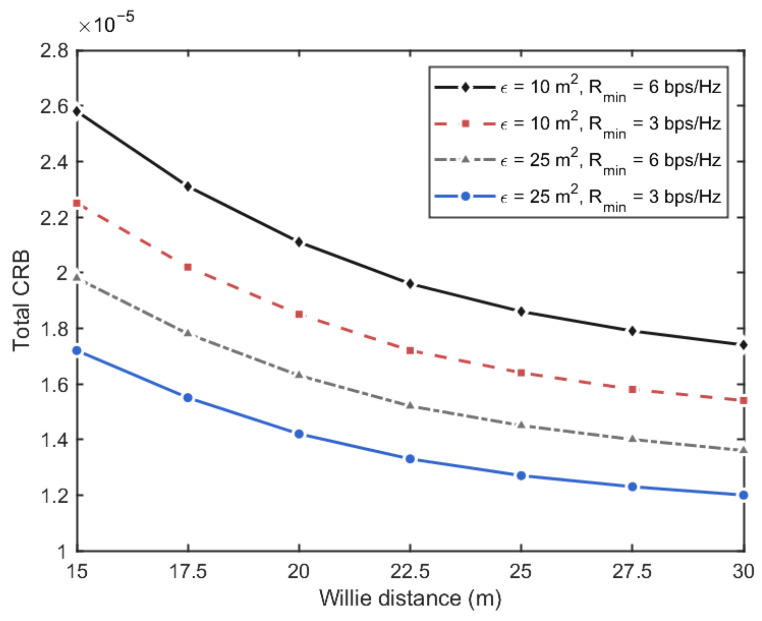
Sensing performance versus Willie distance $r_{w}$ under different covertness constraints ϵ and minimum communication rate requirements $R_{\min}$ of the PU and OUs, where Willie is located along the PU direction with $r_{w}\geq r_{p}$.

Figure 6 illustrates the sensing performance in terms of the Total CRB versus the Willie distance $r_{w}$ under different covertness constraints ϵ and communication rate requirements $R_{\min}$, where the PU location is fixed and Willie is assumed to lie along the same angular direction as the PU with $r_{w}\geq r_{p}$. The minimum communication rate requirements of the PU and OUs are identical and denoted by $R_{\min}$. It is evident that the Total CRB decreases monotonically as $r_{w}$ increases for all considered parameter settings, indicating that the sensing accuracy improves when Willie moves farther away from the PU. This trend reflects the fact that a larger Willie distance relaxes the covertness requirement imposed on the transmit design, thereby allowing the joint sensing and communication beamforming to better favor the sensing objective. Moreover, the curves are more separated when Willie is located near the PU position, showing that the sensing performance is more sensitive to the design constraints in this regime. As $r_{w}$ increases, the curves gradually become flatter and move closer to each other, indicating that the influence of Willie on the overall sensing, communication, and covertness performance diminishes when the warden is sufficiently far from the PU.

### 4.5. Effect of ULA Configuration on Joint Sensing and Communication Performance

**Figure 7:**
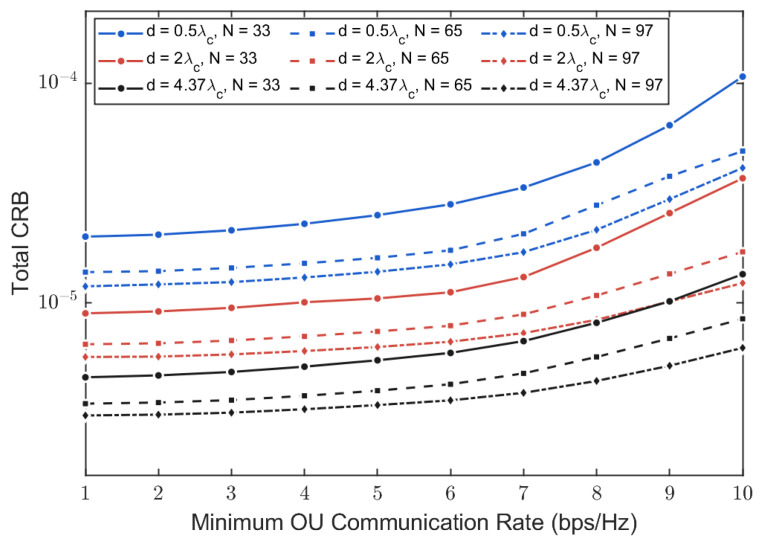
Total CRB versus the minimum OU communication rate under different ULA configurations. Each curve corresponds to a specific combination of the inter-element spacing $d\in \{0.5\;\lambda_{c},\;2\lambda_{c},\;4.37\;\lambda_{c}\}$ and the number of antennas N∈{33,65,97}.

To further investigate the impact of the ULA configuration on sensing performance, we evaluate the Total CRB under different combinations of the antenna number *N* and the inter element spacing *d*. The PU minimum rate requirement is set as 5 bps/Hz, and the vulnerable region area constraint is set as $S_{v}=10\;m^{2}$. We consider N∈{33,65,97} and $d\in \{0.5\;\lambda_{c},\;2\;\lambda_{c},\;4.37\;\lambda_{c}\}$, where $\lambda_{c}$ denotes the carrier wavelength. The results are shown in Figure 7. It can be observed that the Total CRB generally increases as the minimum OU communication rate becomes more stringent, since more power resources are required to satisfy the communication constraint. More importantly, for a fixed antenna number *N*, increasing the inter-element spacing *d* from $0.5\;\lambda_{c}$ to $2\;\lambda_{c}$ and $4.37\;\lambda_{c}$ leads to a decrease in the Total CRB, which indicates an improvement in sensing accuracy. This suggests that the large-spacing ULA configuration adopted in the simulations can more effectively exploit the near-field characteristics for sensing under the considered setup. In addition, for a fixed *d*, increasing *N* also leads to a decrease in the Total CRB, indicating that a larger number of antennas can further improve the sensing accuracy.

## 5. Discussion

This paper investigated covert sensing and communication in a multi-user near-field ISAC system. An optimization framework was developed to minimize the sensing CRB while satisfying the communication rate requirements of the PU and OUs and constraining the vulnerable region area for covert transmission. To solve the resulting nonconvex problem, a hybrid AO-SCA algorithm was designed by combining SDR-based covariance optimization and finite central difference gradient evaluation. Numerical results verified the convergence of the proposed algorithm and showed that the adopted numerical gradient implementation is robust to the tested perturbation parameters. The simulation results also demonstrated the tradeoff among sensing accuracy, communication requirements, and covertness. Specifically, stricter OU rate requirements and smaller vulnerable region constraints increase the Total CRB, indicating a sensing performance loss caused by more stringent communication and covertness constraints. The results further showed that the vulnerable region area can be effectively controlled by the proposed design. When Willie is farther from the PU, the covertness constraint becomes less restrictive, and the sensing performance improves. In addition, increasing the antenna number or the inter-element spacing reduces the Total CRB under the considered simulation settings. A comparison with a fully optimized PU beamformer showed that the MRT-based PU transmission achieves comparable sensing performance in the low-rate region while maintaining a more tractable problem structure. Future work may extend the proposed framework to dynamic scenarios with mobile users, targets, and wardens, where real-time resource allocation and robust vulnerable region control need to be jointly considered. In particular, mobility and practical modeling errors may change the near-field focusing pattern and the resulting vulnerable region boundary, motivating adaptive and robust optimization methods.

## Figures and Tables

**Table 1:** Dominant arithmetic operations in one AO iteration.

Block	Complex Multiplications	Complex Additions
PU update, (Equation (29a–e))	$2N^{2}+N$	$\left(K+2\right)N^{2}$
Each OU update, (Equation (30a–d))	$3N^{2}+N+O\left(N^{3.5}\right)$	$\left(K+2\right)N^{2}+O\left(N^{3.5}\right)$
Sensing update, (Equation (31a–c))	$N^{2}+O\left(N^{3.5}\right)$	$\left(K+1\right)N^{2}+O\left(N^{3.5}\right)$
CRB gradient	$O(N^{4})$	$O(N^{4})$
$S_{v}$ gradient	O(1)	O(1)

**Table 2:** Sensitivity of the AO-SCA algorithm to the perturbation parameters used in numerical gradient estimation when the minimum communication rate of the OUs is 6 bps/Hz, the minimum communication rate of the PU is 5 bps/Hz, and $S_{v}=10\;m^{2}$. The perturbation step δ is dimensionless and applied to the normalized covariance perturbation, while $\Delta P_{p}$ and $\Delta P_{\mathrm{int}}$ are taken in the linear power domain with unit W.

δ	$\Delta P_{p}=\Delta P_{\mathrm{int}}$	Average AO Iterations	Total CRB	Relative Change
0.005	$1\times 10^{-3}$	41	$3.97\times 10^{-6}$	−0.25%
0.01	$1\times 10^{-3}$	26	$3.98\times 10^{-6}$	0.00%
0.02	$1\times 10^{-3}$	18	$4.04\times 10^{-6}$	+1.51%
0.01	$5\times 10^{-4}$	34	$3.96\times 10^{-6}$	−0.50%
0.01	$2\times 10^{-3}$	19	$4.07\times 10^{-6}$	+2.26%

## Data Availability

Data sharing is not applicable to this article.
